# Application of Homograft Valved Conduit in Cardiac Surgery

**DOI:** 10.3389/fcvm.2021.740871

**Published:** 2021-10-12

**Authors:** Yige Huyan, Yuan Chang, Jiangping Song

**Affiliations:** The Cardiomyopathy Research Group at Fuwai Hospital, State Key Laboratory of Cardiovascular Disease, Chinese Academy of Medical Sciences and Peking Union Medical College, Beijing, China

**Keywords:** homograft valved conduit, cardiac surgery, xenograft valved conduit, decellularized valved conduit, tissue engineered substitutes

## Abstract

Valved conduits often correct the blood flow of congenital heart disease by connecting the right ventricle to the pulmonary artery (RV-PA). The homograft valved conduit was invented in the 1960s, but its wide application is limited due to the lack of effective sterilization and preservation methods. Modern cryopreservation prolongs the preservation time of homograft valved conduit, which makes it become the most important treatment at present, and is widely used in Ross and other operations. However, homograft valved conduit has limited biocompatibility and durability and lacks any additional growth capacity. Therefore, decellularized valved conduit has been proposed as an effective improved method, which can reduce immune response and calcification, and has potential growth ability. In addition, as a possible substitute, commercial xenograft valved conduit has certain advantages in clinical application, and tissue engineering artificial valved conduit needs to be further studied.

## Introduction

In tetralogy of Fallot combined with pulmonary atresia or other types of severe cardiac malformations, the use of valved conduits to reconstruct the ventricular outflow tract is required to restore normal hemodynamics. From the 1960s, when valved conduits were first used, improvements in preservation methods have greatly expanded the use of homograft valved conduits in clinical practice. This technology has been rapidly developed at present. The basic research, clinical trials, and follow-up reports on valved conduits have been published continuously, opening up new avenues for the treatment of complex precordial and vascular disease. This article reviews the advances in homograft valved conduit and its clinical applications and provides an outlook on this field.

## Methods

An electronic search in PubMed was performed from inception until 31 April 2021. The search strategy consisted of free and controlled terms for homograft valved conduit. The search was limited to publications in English. Reference lists of previous reviews of the subject as well as the included articles were hand-searched to identify additional eligible studies. Following retrieval of the search results, the title and abstract of the remaining records were screened. Relevant articles and articles of which eligibility could not be assessed properly were selected for full-text assessment. Then select relevant articles to be included in this review.

## The History of Homograft Valved Conduit

In 1966, Ross et al. used homograft valved conduit to connect right ventricular outflow tract and pulmonary artery for the first time. They used valved homograft aorta and pulmonary artery to treat pulmonary atresia, and then gradually expanded to tetralogy of Fallot, double outlet of right ventricle, transposition of great arteries, single ventricle, permanent truncus arteriosus, and many other complex congenital heart diseases. In the 1960s, five cases of pulmonary atresia variant transposition of aorta with occlusion of left ventricular outflow tract were reported, all of which were repaired with valved homograft aorta at the site of pulmonary artery (1, 2). At the Mayo Clinic, there is relatively large early experience in radiation cryopreservation of homograft valved conduit. However, the graft was found to have calcification and rapid degeneration 1–3 years after operation, resulting in severe stenosis (3). Subsequently, several international centers disinfected the homograft valved conduit with antibiotics and preserved them in a tissue medium at 4°C (4, 5). In the experience of a limited number of centers, these conduits performed well in the location of the pulmonary artery. However, the shelf life of this kind of conduits is very short, so it is difficult to become a widely used homograft technology.

**Figure 1:**
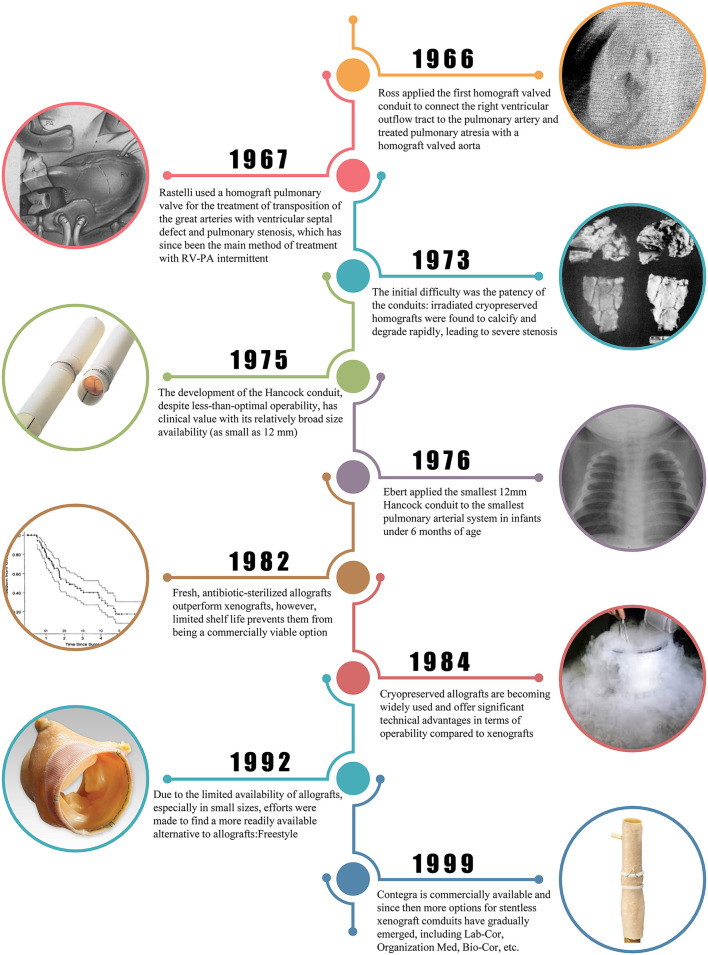
Historical progression since 1966 when Ross first applied homograft valved conduit.

The development of homograft valved conduit preservation methods can be broadly divided into four phases: the first stage (mid-1960s) was the *in situ* operation method, in which fresh homograft valved conduit was obtained under sterile conditions and perform surgical treatment within a few hours. In the second stage (mid-1960s to mid-1970s), chemical reagents or radiation sterilization were used, followed by cryopreservation at −79°C (6). Because sterilization also leads to loss of biological activity of the homograft valve, the valve is prone to degeneration, calcification after surgery, and poor long-term results. In the third stage (mid-1970s to mid-1980s), isolated homograft valved conduit were sterilized in antibiotic solution for 24 h and then stored at a low temperature of 4°C in a nutrient solution containing low concentrations of antibiotics, which maintained their biological activity and short and long-term clinical outcomes, but the maximum storage time was only 6 weeks, which was still unsatisfactory (7). The fourth stage (after the mid-1980s) used antibiotic sterilization, controlled rate cooling, and liquid nitrogen deep cryopreservation (8). This is currently the most widely used and studied method internationally. The main process is to sterilize isolated homograft valved conduit with antibiotic solution at 37°C for 24 h, then put into RPMI1640 medium containing dimethyl sulfoxide (DMSO) and cool down to −80°C at a rate of −1°C/min, and then transfer to liquid nitrogen for deep cryopreservation for long-term storage. Figure 1 illustrates the development of valved conduits, including milestones of homograft valved conduits and some xenograft valved conduit.

## Preservation Methods for Homograft Valved Conduit

The biological activity of homograft valved conduit and the integrity of its tissue structure are the keys to preserve its function. The biological activity of homograft valved conduit refers to the number of living cells it contains and its functional state, while the integrity of tissue structure refers to the composition and structural integrity of the extracellular matrix. Fibroblasts can synthesize extracellular matrix and play an important role in maintaining the stability of valved conduit (9). The increased possibility of thrombosis caused by the loss of endothelial cells will also accelerate the failure of homograft (10). Current research advances in homograft valved conduit preservation methods focus on donor conditions, warm ischemia time, sterilization methods, freezing methods, and rewarming methods (11).

The health status of the donor itself determines the biological activity and the structural integrity of the tissue of the homograft valved conduit. In clinical applications, Sadowski et al. found that valves removed from elderly donors were prone to complications such as degeneration and incomplete closure (12). Verghese et al. noted that homograft valved conduit from individuals after organ or tissue transplantation readily induces an immune response in the recipient and should not be used as a donor (13). Gall et al. quantitatively evaluated the initial activity of homograft valved conduit in normal and cardiac arrest donors by thin-layer radiographic autoradiography, and the initial activity of homograft valved conduit in normal donors was significantly higher (92 ± 2)% than that in cardiac arrest donors (66 ± 3)%, suggesting that the biological activity of homograft valved conduit is significantly reduced after cardiac arrest due to the lack of oxygen and nutrient supply (14). Therefore, to maximize preservation of homograft valved conduit activity prior to operation, it should be removed as soon as possible after donor cardiac arrest, or if the corpse is cryopreserved it can still be collected within 24 h. Donors currently used in clinical practice are generally selected from brain dead or autopsied patients under 45 years of age (6) who have no major vascular disease, valve disease, infectious disease or autoimmune disease in their lifetime. Homograft valved conduit are removed under strict aseptic conditions, placed in saline or Hank's solution containing antibiotics, packed in double-layer sterile bags, and shipped as soon as possible at 4°C for trimming and subsequent processing.

Warm ischemia time is a period of time between when homograft valved conduit loses blood supply after cardiac arrest and when it is taken out and stored at 0–4°C. Mohan et al. found that when warm ischemia time >24 h, homograft valved conduit endothelial cells basically die and fall off, and the synthesis of prostacyclin stops. The collagen fibers in the artery wall are destroyed, and the structural integrity is also destroyed (14). Suh et al. studied the activity of porcine aortic valve fibroblasts under different conditions. Fibroblast activity was (92.25 ± 2.7)% at 2 h of heat ischemia and (57.0 ± 10)% at 24 h of heat ischemia (*P* < 0.05), suggesting a significant decrease in fibroblast activity with increasing warm ischemia time. Suh et al. studied the activity of porcine aortic valve fibroblasts under different conditions. Fibroblast activity was (92.25 ± 2.7)% at 2 h of warm ischemia and (57.0 ± 10)% at 24 h of warm ischemia (*P* < 0.05), suggesting a significant decrease in fibroblast activity with increasing warm ischemia time (15). Burkert et al. applied scanning electron microscopy to observe the effects of different warm ischemia time on human aortic valves. When the valve was left at room temperature for 12 h, the continuity between valve endothelial cells was disrupted, some endothelial cells were shed into the lumen, and endothelial cell loss occurred. When warm ischemia time reached 48 h, the vascular endothelial cells were completely shed, while the vessel wall structure was destroyed. By comparing the degree of damage to the homograft valved conduit by different warm ischemia time, it is recommended that warm ischemia time should be controlled within 24 h as much as possible (16). Stemper et al. found that vascular tissue mechanics were significantly reduced after more than 24 h compared to fresh vessels, even after storage at 4°C (17). The above studies provide an objective experimental basis for clinical control of homograft valved conduit with a warm ischemia time of <24 h.

The classic antibiotic solution formulation is penicillin 50 U/ml, streptomycin 50 μg/ml, and amphotericin B 10 μg/ml. However, Strickett et al. found that amphotericin B and streptomycin are highly cytotoxic and can significantly reduce the activity of valvular fibroblasts (17). Yu Haibin et al. used cefoxitin 240 mg/L, polymyxin B 100 mg/L, lincomycin 120 mg/L, and vancomycin 50 mg/L for sterilization treatment, which ensured sterilization and avoided the effect of streptomycin and amphotericin B on cell activity. Jashari et al. found that the removal of cefoxitin and the use of lower concentrations of lincomycin, polymyxin B, and vancomycin had no significant effect on cell survival and the sterilization effect was reliable (18). Since antibiotics are sterilized primarily during the bacterial multiplication phase, antibiotic sterilization activity is greatest at 37°C. However, the normal metabolic rate of valve tissue at 37°C inevitably leads to inactivation of valve tissue cells due to hypoxia and nutrient deficiency. The 4°C incubation sterilization protocol was first proposed by Kinklin et al. found that valve sterilization at 4°C for 20 h resulted in a slower metabolic rate and increased tolerance to lack of oxygen and nutrients, but a positive sterilization effect (19).

Currently, the main clinical methods used are incubation sterilization with antibiotic solution, controlled rate cooling, and liquid nitrogen freezing, as proposed by O'Brien. The main mechanism is that the deep cryogenic environment can interfere with cell metabolism and inhibit the biochemical activities of the organism. Brockbank et al. compared the protein synthesis function of homograft valved conduit fibroblasts preserved by different methods. After 2 years of preservation by liquid nitrogen freezing, the fibroblasts still had protein synthesis function, and the protein synthesis function of freeze-dried fibroblasts gradually decreased with time. The protein synthesis function of fibroblasts preserved at 4°C for 2 weeks was only 15% of that of the liquid nitrogen preservation group (20). Liquid nitrogen freezing is effective in preserving tissues and organs, but cryoinjury still exists. Dimethyl sulfoxide is currently the most commonly used homograft valved conduit liquid nitrogen cryoprotectant. It can rapidly penetrate cell membranes, lower the freezing point, slow down the cryopreservation process, increase the intracellular ion concentration, and reduce intracellular ice crystal formation, thereby reducing cellular damage. However, high concentrations of DMSO are inherently cytotoxic and can cause osmotic damage. The optimal concentration of DMSO in cryopreservation solution is currently considered to be 15% (21). Wosteman et al. found that replacing sodium salts with vitamin B complexes in cryopreservation solutions prevented DMSO from exerting its toxic effects (22). Mazur et al. found that during rapid freezing, water molecules in the intracellular fluid do not have time to penetrate outside the cell and form ice crystals inside the cell, which disrupt the ultrastructure of the cell and lead to cell death. During slow freezing, cells are exposed to hyperosmotic extracellular fluid and toxic antifreeze for too long, which may also lead to cell death (23). Therefore, the cooling rate is very important to maintain the biological activity of cells. The use of a program-controlled hypothermia instrument to control homograft valved conduit cooling at a relatively stable and reasonable rate can minimize cryo-damage. −1°C/min was found by Vander et al. to be the best cooling rate to maximize fibroblast activity (24). In recent years, the concept of phase change point has received increasing attention in the process of programmed cooling. The phase change point is the temperature point at which the sample transforms from liquid to solid during the freezing process, and a large amount of melt heat is released during the morphological transformation, causing the local sample to condense, and then increase in temperature and melt, and then condense again. During this process, the cells are susceptible to repeated damage by ice crystals, which affects the preservation effect of homograft valved conduit. To overcome the phase transition heat, increasing the cooling rate near the phase transition point to prevent the temperature increase during the phase transition and decreasing the cooling rate after complete phase transition to continue cooling at −1°C/min can reduce the damage to the homograft valved conduit from the phase transition. The phase transition point of human homograft aortic valved conduits is −4 to −6°C. During this period, increasing the cooling rate to −4°C/min instead of −1°C/min can effectively reduce cell damage.

**Figure 2:**
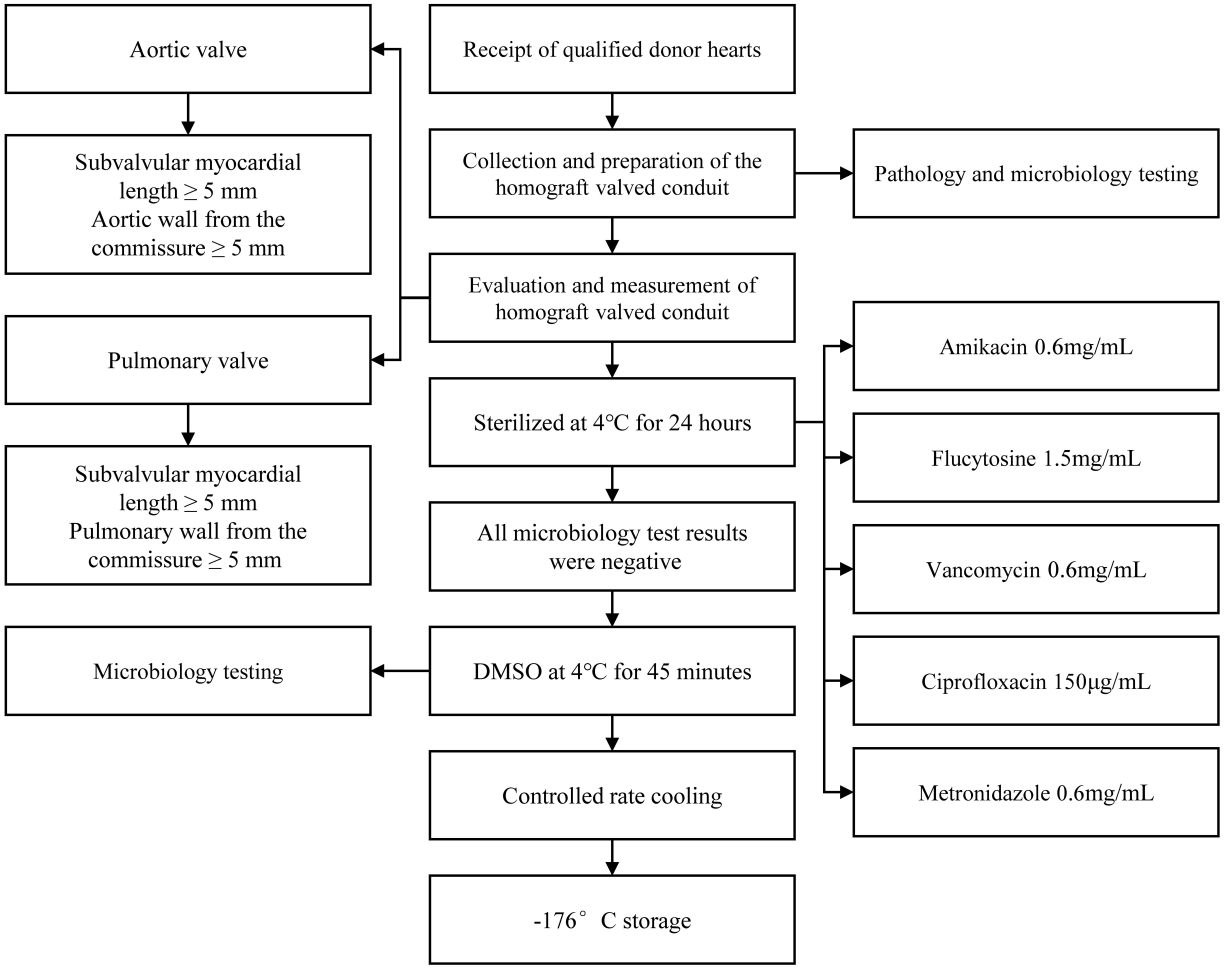
Specific handling of homograft valved conduit from donor acquisition to deep cryogenic storage. It is important to note that (1) extra care must be taken throughout the processing and repeated microbiological tests must be performed to ensure complete sterile processing and storage, as any contamination could be catastrophic to the recipient; (2) the sterilization recipe in the figure is the protocol of Berlin, Germany, and different sterilization protocols may exist in other institutions, and there is no uniform protocol yet, sterilization to meet microbiological testing standards is a feasible solution.

Liquid nitrogen cryopreserved homograft valved conduit needs to be thawed and resuscitated before it can be used in the clinic. Rapid rewarming in a 42°C water bath is now commonly accepted in clinical practice. Rapid rewarming is particularly important for frozen cells. For cells containing ice crystals, rapid rewarming can limit the phenomenon of migratory recrystallization within the cells and reduce the damage caused by repeated crystallization, thus improving cell survival for cytoprotection. Since DMSO is toxic to cells at room temperature, when homograft valved conduit is rewarmed and melted, it should be quickly removed and replaced by “graded series rinsing” to reduce its damage to cells. This preservation method not only retains the biological activity of the valved conduit, but also retains a certain degree of antigenicity. Most patients implanted with valved conduit have humoral immunity against human leukocyte antigen (HLA) (25). It has been found that donor-derived dendritic cells and endothelial cells expressing HLA-II molecules in valved conduits can directly present antigens and activate receptor immunity (26, 27). Other studies have shown that there are mild to moderate mononuclear inflammatory cell infiltration composed of T cells and macrophages in valvular tissue (28). Figure 2 summarizes the processing flow of homograft valved conduit preservation method (29).

## Clinical Application of Homograft Valved Conduit

One of the tasks frequently faced by congenital heart surgeons is the reconstruction of the right ventricular outflow tract, i.e., the creation of a channel between the right ventricle and the pulmonary artery. Depending on the etiology of the pathological changes of the pulmonary outflow tract, it can be classified as tetralogy of Fallot combined with pulmonary atresia, congenital arterial trunk right ventricular outflow tract or pulmonary artery agenesis, transposition of the great arteries with ventricular septal defect and pulmonary stenosis, corrected transposition of the great arteries with pulmonary stenosis, complex right ventricular double outlet, and Ross procedure of medical origin (30).

The Ross procedure, using the autograft pulmonary valve to replace the diseased aortic valve and a non-autograft valved conduit to replace the pulmonary valve, has become the procedure of choice for young people with irreparable aortic root disease. There are two main techniques for Ross procedure: the subcoronary and root replacement techniques. The subcoronary technique can be technically challenging because the aortic and pulmonary roots often have different dimensions and commissural distribution. Therefore, many surgeons use the full root replacement technique for Ross procedures (31, 32). The Ross procedure is reserved for patients with non-repairable, non-spareable aortic valves. Otherwise, isolated aortic valve repair or valve-sparing root replacement should be chosen. Ideal candidates for the Ross procedure are young or middle-aged (<50 years old). Patients presenting with mechanical or bioprosthetic aortic valve dysfunction and patients with active endocarditis also appear to be appropriate candidates for surgery. Athletes are also suitable because of the absence of anticoagulation and extreme physiological and hemodynamic consequences (33). Chronic complications after the Ross surgery including aortic valve insufficiency, right ventricular outflow tract obstruction, autograft aortic valve dilatation, and homograft pulmonary valve stenosis. Potential long-term failure of both valves (aortic and pulmonary) has been considered the Achilles' heel of the Ross procedure (34). Reece et al. (35) reported a three-fold increase in mortality with the Ross procedure compared with conventional AVR. However, the Ross procedure is more complex than standard AVR. Some of the steps required in the Ross procedure—but not in conventional AVR—include dissection of the aortic root, mobilization of the coronary arteries, harvesting of the pulmonary autograft, proximal autograft anastomosis, coronary artery reimplantation, and pulmonary homograft implantation. Each of these steps increases the risk of the procedure (36). Importantly, the increase in mortality was mainly observed in low-volume centers. Therefore, for the complex surgery, these suboptimal outcomes are not surprising (37). In contrast, several experienced high-volume centers have demonstrated that the mortality rate for performing Ross surgery is similar to that of conventional AVR (31). This emphasizes the importance of the surgeon expertise and adequate surgical volume to achieve excellent outcomes in Ross surgery.

There are some findings in echocardiograms at follow-up after right ventricular outflow tract reconstruction. Pulmonary autograft dilatation is common in adults after Ross surgery, which may be the reason for reoperation. This dilatation develops over time and is usually accompanied by a dilatation of the native aorta. In a study of 71 patients with a median follow-up of 8.9 years (38), the proportion of patients with enlarged autografts and proximal ascending aorta was 13 and 16%, increasing to 33 and 44%, respectively. A retrospective multicenter international cohort study monitored the peak transvalvular gradients and regurgitation grade. Female sex, tricuspid natural aortic valve, and higher preoperative gradients were significantly associated with higher autograft gradients. Female sex was significantly associated with lower gradients of homografts, but also increased the likelihood of significant regurgitation. In contrast, the use of homografts rather than bioprosthesis and older donor age was associated with a lower likelihood of significant regurgitation (39). Another study showed that age and homograft diameter at the time of operation was also associated with valve function (40).

Homograft pulmonary artery is the best option for reconstructing RVOT, has good durability, and is not necessary for adults to undergo valve replacement again. Alexander et al. (41) evaluated 741 adult patients who underwent the Ross procedure. Right ventricular outflow tract reconstruction included 175 (23.6%) homograft pulmonary arteries, 561 (75.7%) porcine or bovine grafts, and 5 (0.7%) polytetrafluoroethylene duct reconstructions. The mean follow-up time was 5.8 ± 2.2 years. The 10-year survival rate was 90.7%, comparable to the age- and sex-matched general population. The 5- and 10-year graft reoperation rates were 94.1 and 88.3%, respectively. The probability of not reoperation at 10 years for allografts, pericardial xenografts treated with bicycloxide or glutaraldehyde, and porcine aortic root grafts was 100, 94.4, 82.7, and 80.6%, respectively. Jamie et al. (40) reported the implantation of 701 homograft pulmonary valved conduits in 604 patients. At 25 years of follow-up, the survival rate was 84 ± 4% and the valve replacement rate was 56 ± 6%. The probability of not requiring valve replacement after 15 years was 28 ± 14% in patients younger than 1 year, 59 ± 8% in patients 1–18 years, and 82 ± 5% in patients older than 18 years. Patients reported lower levels of physical strength and general health compared with the sex- and age-matched Dutch population, but significantly less physical pain. Pieter et al. (42) reported a total of 133 homograft valved conduits implanted in 126 patients with tetralogy of Fallot, including 126 pulmonary valved conduits and 7 aortic valved conduits. At long-term follow-up, the homograft conduits functioned satisfactorily in the pulmonary artery location. Clinical study data from Parth et al. (43) showed good mid-term durability of the homograft pulmonary valved conduits when used for right ventricular outflow tract reconstruction for the Ross procedure. At present, the main clinical application is pulmonary valved conduit, while aortic valved conduit is less. The reason may be that atherosclerosis and calcification are easy to involve the aorta, so the source of this kind of valved conduit is relatively limited. Anatomical, histological and biomechanical tests *in vitro* show that both conduits can meet the needs of clinical application (44, 45), but there is a lack of randomized controlled trials to compare the two conduits. The results of retrospective clinical studies are controversial. Some studies do not show significant differences (46, 47), while other studies have shown that the survival rate of pulmonary valved conduit is higher than that of aortic valved conduit (48, 49).

Homograft pulmonary arteries perform well in adult cardiac surgery, but have no significant clinical advantage in pediatric cardiac surgery due to limited sources and size. John et al. (50) reported 40 cases of Rastelli's procedure. There were 32 cases of right ventricular outflow tract obstruction and eight cases of atresia. Follow-up was obtained in all but one case with a mean of (8.6 ± 5.6) years. The right ventricular outflow tract was reconstructed using homograft conduits (25 cases), bovine jugular vein (BJV) (eight cases), valveless polyester conduits (five cases), and porcine valved conduits (two cases). The conduit-free replacement rates at 5, 10, 15, and 20 years were 86, 74, 63, and 59%, respectively. Multifactorial analysis showed that younger intraoperative age was a risk factor for conduit replacement (*p* < 0.001). Early performance of small BJVs may be more favorable than homografts. Andrew et al. (51) compared the results of initial placement of smaller diameter BJVs (12–14 mm) and homograft pulmonary valved conduits (10–15 mm) in children younger than 2.84 years (mean age 8.4 ± 8.5 months). Eighty-four children received BJV (*n* = 51) or homograft pulmonary artery (*n* = 32) conduit placement. Early and late mortality rates were similar (BJV 80%; pulmonary artery 88%; *P* = 0.55). None of the deaths were graft-related. The degree of functional recovery was significantly better in the BJV group at 5 and 10 years after surgery (85 and 90% in the bovine jugular group; 71 and 24% in the pulmonary artery group, respectively; *P* < 0.05). In the pulmonary valved conduit cohort, the probability of conduit failure at 5 and 10 years was higher (85 and 67% in the BJV group; 75 and 45% in the pulmonary artery group, respectively; *P* = 0.06). The probability of avoiding reoperation was significantly better in patients with implanted BJV conduits than in the pulmonary artery group (85 and 47%, respectively; *P* < 0.001). Although the early performance of BJVs may be superior to that of homografts, the incidence of infective endocarditis after right ventricle-pulmonary artery conduit implantation suggests that conduits of bovine origin are more susceptible to endocarditis (52, 53), which may be associated with suboptimal hemodynamics (54) and thrombus apposition at the basis of conduit valve sinus (55). Meanwhile, Mercer et al. (56) demonstrated that polytetrafluoroethylene conduits with mitral valves are ideal for right ventricular outflow tract reconstruction in children under 2 years of age. Their availability, low cost, and lack of potential sensitization make them an attractive alternative to homograft conduits. Based on the above problems, we can consider using bicuspidalized to reduce the size of valved conduit in infants (57, 58). A retrospective study analyzed 93 conduits which are <20 mm implanted for more than 23 years, including 40 standard pulmonary conduits, 12 standard aortic conduits, 17 bicuspidalized conduits and 24 xenografts (59). The average follow-up time was 7.6 ± 5.9 years. The percentage of patients without structural valve degeneration for 10 years was 47 ± 6%, including 68 ± 8% of pulmonary conduits, 42 ± 16% of bicuspidalized conduits, 31 ± 15% of aortic conduits, and 20 ± 9% of xenografts (log rank *P* < 0.001). Therefore, the appropriate size of pulmonary valved conduit is still the most durable choice for right ventricular outflow tract in children. However, when there is no available size, the mid-term follow-up results show that bicuspidalization can provide an effective alternative compared to xenograft. Other studies have also shown that bicuspidalized valved conduit seems to be a feasible option to solve the problem of limited supply of small size allografts, with acceptable survival rate and reoperation rate (60–62).

Homograft valved femoral veins with both smaller size and less risk of infection have shown good potential in pediatric cardiac surgery. The Yasui procedure is indicated for ventricular septal defects with interrupted aortic arch and associated subaortic stenosis. Manan et al. (63) described a modified Yasui procedure in which aortic reconstruction is simplified using a valveless homograft femoral vein connecting the pulmonary artery to the descending aorta. The homograft femoral vein is laterally anastomosed to the ascending aorta to complete the new aortic reconstruction. The anomalous opening from the left ventricle to the pulmonary artery is repaired with a patch, followed by restoration of continuity of the right ventricular outflow tract using a valved homograft femoral vein. This eponymous repair technique for aortic arch disruption and severe left ventricular outflow tract obstruction using a homograft valved femoral vein was first described in a case report by Yasui. According to Kumar et al. (64), the homograft femoral vein is safe as a conduit from the right ventricle to the pulmonary artery (RV-PA) in the Norwood procedure, and echocardiography shows good pulmonary artery growth and preserved ventricular function. Ofer et al. (65) reported that homograft valved femoral veins have similar short- and medium-term performance to homograft aorta or pulmonary artery in reconstructing the right ventricular outflow tract and are an attractive alternative to the smaller conduits used in neonates and infants. Histopathological evaluation showed that the valve maintained most of its function, and venous wall remodeling included only mild inflammation and calcification (66). A summary of clinical trials of homograft valved conduits over the past decade is shown in Table 1.

**Table 1:** Clinical trial data of homograft valved conduits in the last decade.

**Period**	**Number of patients**	**Number of patients receiving homograft valved conduit**	**Mean age (years)**	**Mean follow-up time (years)**	**Surgical procedure**	**Avoiding of valve replacement rates**	**Conference**
1998–2014	741	175	47.4	5.8	Ross	10 years: 100%	(41)
1986–2017	604	604	19.5	11.4	RVOT reconstruction	25 years: 76%	(40)
1987–2009	126	126	27.8	8.1	TOF	10years: 83%	(42)
1998–2016	30	15	3.3	6.8	Ross	5 years: 87%	(43)
1988–2008	40	25	4	8.6	Rastelli	10 years: 74%	(50)
1998–2009	84	32	0.7	5.9	RVOT reconstruction	10 years: 55%	(51)
2004–2014	54	26	0.35	2.7	RVOT reconstruction	End of follow-up: 48%	(56)

Homograft valved conduit has good biological properties and clinical results, but its durability is not very satisfactory (67). Age ≤ 9 years old is one of the main factors affecting durability (68, 69). Small size congduits (<19 mm) are also associated with decreased graft survival (70, 71). The type of conduits is considered to be one of the most important factors in long-term durability, for example, the survival rate of pulmonary conduit is higher than that of aortic conduit (70, 72). Baltivala et al. found that the graft survival rate of patients with a history of transplantation was worse than that of other patients (73). The relationship between bioactivity and durability of homograft valved conduit is still controversial. Fibroblasts living in the graft can reshape and reconstruct collagen structure and extracellular matrix, thus enhancing durability (74). However, these unevenly distributed fibroblasts may have phenotypic change and abnormal biological behavior due to immune response or environmental changes. Valve distortion or loss of elasticity caused by punctate or extensive hypertrophy, resulting in easy to rupture (75). The existence of endothelial cells reduces the possibility of thrombosis, but at the same time increases the antigenicity of the graft. The immune response is related to the early degeneration and dysfunction of the graft (76). Other factors related to the preservation process, including donor conditions, WIT, etc. will also affect the durability of valved conduits.

## Xenograft Valved Conduits and Other Possible Alternatives

Xenograft valved conduit can prolong the time of freedom from operation and has a good long-term survival rate (77). The Medtronic Freestyle (Minneapolis, MN) is a glutaraldehyde fixed stentless porcine aortic valved conduit. Because of its standard availability from 19 to 29 mm in diameter and potential for longer valve function compared to homograft, several authors have published a series of studies on the Freestyle implantation, primarily for phase II RVOT reconstruction (78, 79). Early results performed satisfactorily with minimal pressure gradients and little or no valvular regurgitation. In the published reports, the follow-up time was <3 years. Long-term data for this conduit have not been published. Although the Freestyle performs well *in situ*, in most adult patients, the Freestyle is usually not long enough to reach the pulmonary artery from the free wall of the right ventricle, which can be addressed by anastomosing the Freestyle graft to the pulmonary artery and then connecting the Freestyle proximally to the right ventricle with an expanded large-bore polytetrafluoroethylene or braided polyester conduit. Ezelsoy et al. (80) reported 77 patients with ascending aortic aneurysm combined with aortic valve insufficiency who underwent total root replacement using Freestyle. The median follow-up was 11.2 years. The probability of freedom from aortic valve reoperation at 5 and 10 years was 97.4 ± 1.2 and 93.4 ± 4.9%, respectively. Freestyle demonstrated favorable clinical outcomes in terms of survival and structural degeneration of the valve. Freestyle is a viable option for patients who are undergoing bioprosthetic aortic valve replacement and expect long-term durability. Mehdiani et al. found that the mid-term clinical and hemodynamic results of BioIntegral composite biological and stentless Freestyle conduits in patients undergoing full aortic root replacement are similar, but the simplified implantation technique of BioIntegral shortens the time of cardiopulmonary bypass and operation (81). The RVOT Elan conduit consists of a stentless valve (Vascutek Elan porcine stentless heart valve) sewn into a vascular graft (Vascutek Biblex Valsalva), showed good hemodynamic performance during an one-year short-term follow-up (82). Another type of diepoxy-treated porcine aortic conduit showed a higher risk of calcification during follow-up (83).

Despite satisfactory early outcome data for porcine-derived valved conduits, manufacturers are now focusing on another xenograft conduit for pediatric RVOT reconstruction, the valved BJV. Originally developed by VenPro (Irvine, CA) and currently owned and marketed by Medtronic, ConIntegra is a glutaraldehyde-fixed BJV conduit for RVOT reconstruction conduit option. Although several centers have reported that this conduit is more clinically beneficial than homograft (84, 85). However, these data are rather preliminary due to the published mean follow-up period of 12 months or less. At the same time, TakashiKido et al. have shown that early conduit dysfunction can still be caused by more than mild pulmonary hypertension and lower body weight during surgery of <3.0 kg (86). In addition, one center reported significant early fibrous epidermis formation at the distal anastomosis, as well as significant dilation and regurgitation of the conduit in the case of pulmonary hypertension or distal obstruction of the aforementioned distal anastomosis (87). Some studies have shown that BJV conduits have good medium-term durability, but have not been compared with the homograft or other types of conduits (88, 89). Therefore, this conduit will require further observation at longer follow-up times to clarify the differences in performance compared to equivalent sized cryopreserved homograft (43). Alessandro et al. (90) suggests that the 12 mm ConIntegra is an effective alternative to small homogaft valved conduit in the neonatal patient population.

In addition to xenograft valved conduits, synthetic material conduits or conduits containing part of synthetic materials are also gradually being developed for clinical use. The mid-term results of a multicenter clinical trial of simplified tricuspid polytetrafluoroethylene valved conduit show that it has an acceptable functional outcome and service life, freedom from conduit dysfunction was 58.5% at 5 years (91). KONECT RESILIA aortic valved conduit (Edwards Lifesciences, Irvine, Calif) is a noval type of bovine pericardial valved conduit, preassembled with the PERIMOUNT Magna Ease valve and RESILIA tissue with stent. This kind of conduit improves the anti-calcification performance and has dry storage capacity. *In vitro* experiments show that the simulation of sinuses of valsalva structure improves the hemodynamic performance (92).

Porcine small intestinal submucosa extracellular matrix (CorMatrix; CorMatrix Cardiovascular, Rosewell, GA) is a relatively novel tissue substitute. Preclinical experiments applied to pig models show that this biodegradable conduit cannot be reconstructed in a structured and anatomical in the arterial environment. Fibrosis, scarring, and calcification begin at 4 months, and chronic inflammation persists (93). There are also no satisfactory results in the sheep models (94). PTFE valved conduits show excellent hemodynamic performance in preclinical *in vitro* and *in vivo* experiments (95–97). The results of mid-term clinical trials showed less need for further intervention and severe valvular dysfunction (98, 99), especially in valvular regurgitation (100). And the failure incidence of this conduit is lower than that of autologous pericardial valved conduit operation (101). A clinical study involving 502 patients also showed satisfactory long-term results, freedom from conduit explantation was 89.0% at 10 years (102). Histopathological analysis showed that protein infiltration into the valve was thought to be the cause of future calcification and subsequent stenosis failure. Modification of polytetrafluoroethylene to prevent protein infiltration may help to improve the durability of the pipeline (103).

## Advantages and Disadvantages of Homograft and Xenograft Valved Conduit

Although homograft valved conduit was used as early as the 1960s, its widespread use was limited by the lack of effective sterilization and preservation methods (1). Early methods of dealing with homograft valved conduit led to early calcification and progressive stenosis. Current storage methods, deep cryopreservation, delay the onset of these problems, but they still occur in the medium to long term (104). Despite this, compared with other types of valved conduits, homograft valved conduits still have the advantages of biological activity and tissue structural integrity, ease of use, good hemodynamics, intact valve function, long valve life, and no need for anticoagulation. The patency of the conduit after operation is excellent and basically conforms to the anatomical structure of the ventricular outflow tract, corrects cardiac malformations anatomically and hemodynamically, facilitates the recovery of cardiac function, and is less prone to heart failure. The homograft valved conduit has good resistance to infection, and complications caused by implantation such as bacterial endocarditis and hemolysis are rare, but its access is limited.

The BJV graft was introduced in 1999 as an option, which contains a trilobular venous valve (105). Preservation with glutaraldehyde solution under vacuum conditions preserves the flexibility of the valve (106). Because a variety of sizes are readily available, these tubes are more commonly used in clinical practice. The good valvular performance of BJV may be associated with an enlarged leaflet alignment area and reduced sensitivity to deformation of surrounding structures, including the right ventricle, aorta, and chest wall. BJV is associated with distal anastomotic stenosis and a higher incidence of endocarditis compared with homograft (107–112). The Hancock, a stented polyester conduit with porcine valve (113), can also be used in adult cardiac surgery. However, in pediatric applications, calcification as well as poor intraoperative properties due to the polyester conduit, make the Hancock not a common choice.

Current homograft and xenograft conduits lack any capacity for additional growth. As children grow, the fixed size of the conduit means that there is a progressive size mismatch between the patient and the conduit, which necessitates future reoperation. Typically, a new conduit is required approximately 4–5 years after the initial conduit placement in an infant or young child (110). Critically, studies reporting the time of reoperation often combine patients with very different anatomic features, thereby confounding the analysis of outcomes. For example, placement of an RV-PA conduit in pulmonary atresia involves a non-*in situ* and tortuous pathway that allows blood to flow anteriorly from the right ventricle, across the surface of the heart, and then backward to the pulmonary artery. In contrast, placement of the RV-PA conduit in Ross procedure is essentially *in situ* placement of the implant. This may have less turbulence and energy loss than in patients with pulmonary artery atresia, factors that may affect the durability of the conduit.

In addition to body growth, a variety of other problems can lead to conduit failure. These include distal anastomotic stenosis, sternal compression, aneurysm formation at the proximal anastomotic enlargement site, valve stenosis (114), and donor-specific and non-specific immune responses. A study has shown that both homograft and xenograft can trigger an immune response in the recipient, leading to calcification, endothelial thickening or vascular membrane formation, stenosis, and deterioration of valve function (115).

## Possible Future Directions for Improvement

Cryopreserved homograft valved conduit has low clotting activity, good hemodynamic properties, and high resistance to infection, but cannot grow and develop in the recipient. Because cryopreserved homograft valved conduits carry donor cells that express relevant antigens, these cells can cause adverse host immune responses and lead to calcification, which in turn may require reoperation in young patients who receive a homograft valved conduit. Therefore, removal of the cellular component of the homograft valved conduit prior to implantation may reduce the immune response and calcification (116). In addition, removal of donor cells may encourage the proliferation of the cells with the ability to repair and remodel in receptor, thereby overcoming the limitations of degeneration of the homograft valved conduit over time and creating a valve with growth potential in younger patients, thus eliminating the need for reoperation. Decellularized valved conduits demonstrate almost complete removal of cells and cellular components by histological and immunocytochemical analysis without corresponding changes in biomechanics *in vitro* (117). Previous studies have shown that the decellularization process does not affect the *in vivo* performance of homograft valved conduit, and good clinical performance in the short to medium term has been reported for decellularized pulmonary valves, with similar (118–122) or better (123–125) outcomes than conventional deep cryopreserved homograft in adults and children at a mean follow-up of 4 years. However, longer-term data are needed to determine whether they can reduce or eliminate the need for reoperation (126).

SynerGraft (CryoLife Inc., Kennesaw, GA) decellularized pulmonary allograft (SGDPA) is an alternative to standard cryopreserved allograft (SCA) Ross aortic valve replacement. John et al. (127) reported 29 patients underwent SGDPA and 34 underwent SCA during Ross aortic valve replacement. Early clinical and hemodynamic outcomes were favorable but not significantly different from SCA. There were no early or late deaths or major pathologic events during a mean follow-up of 4.9 ± 2.7 years. None of the patients required reoperation. No deterioration in conduit or valve function was seen in the SGDPA group. Several patients with SCA developed mild regurgitation, and one patient developed moderate regurgitation. Thus, the SynerGraft technique may provide a more durable option for patients requiring right ventricular outflow tract reconstruction. Mark et al. (124) also suggests that the medium-term performance of decellularized cryopreserved homograft may be superior to that of standard cryopreserved homograft. Decellularized aortic homograft can withstand the stresses of the body circulation, provide a good effective orifice area, and may be an alternative graft for aortic valve replacement in younger patients. Enlarged aortic root replacement using decellularized aortic homograft is a further option for aortic valve disease associated with dilatation of the ascending aorta, as it avoids the use of any prosthetic material. Igor et al. (128) reported the replacement of the dilated ascending aorta with decellularized aortic homograft in 18 patients with enlarged aortic root replacement. None of the grafts had any degree of dilatation observed during the relatively short follow-up period (mean 2.0 ± 1.8 years, maximum 7.6 years). Intraoperative and postoperative histological analysis of the grafts showed no calcification and revealed extensive recellularization and no inflammation.

Reconstruction of the right ventricular outflow tract in children with decellularized homograft has a low incidence of structural deterioration of the valve and conduit failure. Francisco et al. (129) reported 59 patients who underwent decellularized allograft RVOT reconstruction. The most common procedure was the Ross procedure (34%) and the mean follow-up was 5.4 years. Structural valve deterioration occurred in 13 patients, including five with stenosis and eight with valvular insufficiency. The incidence of no structural valve deterioration from any cause 8 years after surgery was 64.9%. During the follow-up period, CT scans showed no or minimal calcification of the conduit. Grauss et al. (130) believes that decellularization techniques cause much less damage to the valve than recipient's immune response. *In vitro* removal of live cells from cryopreserved homograft may reduce the probability of graft failure. Implantation of autologous or major histocompatibility complex-matched donor endothelial cells would be necessary to reduce damage due to lack of blood-tissue barrier. Willem et al. (131) found that encapsulation of decellularized homograft with FN/SDF-1α prevented cryopreserved heart valve-mediated immune responses, conduit calcification and vascular scar formation, and stimulated reendothelialization.

Decellularization as a promising improvement method not only improves the performance of homografts, but decellularized xenografts also perform well in clinical practice. Decellularized porcine pulmonary artery valved conduits have excellent potential as a substitute for right ventricular outflow tract reconstruction. Ji Luo et al. (132) developed a method for decellularization of porcine pulmonary arterial with minimal effects on biomechanical, hydrodynamic, and leaflet dynamics properties, and immunohistochemical labeling and antibody uptake assays confirmed the lack of α-gal epitopes in decellularized porcine pulmonary artery valve and conduit. *In vitro* biocompatibility studies demonstrated no cytotoxicity in decellularized pulmonary artery valve and conduit. Decellularized bovine pericardial valves also exhibited potent anti-calcification effects in a sheep circulating model (133). However, study by Higuita et al. has shown that the commonly used SDS acellular method may change or eliminate ECM protein, and will lead to the destruction of valve function, while the use of antigen removal tissue treatment can retain the subtle ECM structure and composition of natural tissue, thus preserving the function of natural valve (134). Therefore, the best acellular method needs to be further explored in the field of tissue engineering. Table 2 compares in detail the advantages and disadvantages of different types of valved conduit.

**Table 2:** Advantages and disadvantages of the three types of valved conduits in current clinical application.

**Type of valved conduit**	**Advantages**	**Disadvantages**
Homograft valved conduit	Good hemodynamic and valvular performance, better medium- to long-term clinical outcomes than xenograft valved conduits, relatively low reoperate rates	Restricted source especially in small sizes, presence of some antigenicity that may lead to rejection and early calcification of the conduit especially in pediatric patients, no potential for *in vivo* growth
Xenograft valved conduit	The 12–22 mm size with unlimited sources provides a wide range of applications in pediatric patients	Risk of thrombosis, higher risk of postoperative infection than homograft valved conduit, no potential for *in vivo* growth
SynerGraft decellularized pulmonary allograft	Minimizing rejection and calcification and improving long-term durability, *in vivo* growth potential, current clinical results show that the function and longevity of the conduit is no less than homograft valved conduit and with more significant advantages in pediatric patients	The available evidence is not sufficient to demonstrate the advantages of the long-term effects of decellularized valved conduit and further long-term follow-up is needed

## Prospect

Tissue engineering is an emerging technology with the potential to create implants that can provide structural and mechanical support and are biologically functional. Ideally, grafts with good biocompatibility, the ability to grow in proportion to host tissue growth, and minimal clinical complications will be produced, thus eliminating the need for further surgery. Considering the specific and highly variable cardiac anatomy of patients with congenital heart disease, significant research challenges remain in the design and fabrication of well-durable valved conduits.

Artificial conduit biomaterials need to mimic the mechanical properties of natural arteries and their biomechanical behavior such as hemodynamics while possessing good biocompatibility to avoid clinical complications. In addition, the human arterial structure consists of cytoskeletal proteins such as collagen and elastin deposited in a directional manner, which ensures that natural vessels exhibit complex anisotropic mechanical behavior (135). For example, arterial walls exhibit non-linear elastic properties under stress and hysteresis behavior in response to changes in circulating pressure (136). The key point is that none of the currently available tissue engineering valved conduits has the same material properties as natural tissues, especially anisotropic properties. Computational fluid dynamics can be used to optimize the geometric design of valved conduits and tissue engineering materials. Therefore, fluid mechanics and structural calculation models will continue to play an important role in optimizing the design and manufacturing of patient-specific conduit.

With the development of manufacturing technology, the use of tissue engineering technology to construct artificial valved conduits is under continuous exploration. Traditional 3D porous biodegradable scaffolds planted with cells usually lack the ability of cell differentiation and function, and cannot fully reconstruct the characteristics of natural tissues (137). The advent of technologies such as electrospinning and 3D bioprinting has led to the creation of more complex designs that mimic valved conduit (138). Poly(lactic acid-caprolactone) [P(LA-CL)]-reinforced electrospinning polyglycolic acid (PGA) mesh has been used as a promising composite scaffold and were able to survive in patients, but some developed conduit stenosis, which may be related to the acidic environment caused by the degradation product lactic acid (139). Electrospinning nanofiber grafts are made from composite materials such as polyethylene glycol dimethacrylate, PLA, PCL, and polyvinyl alcohol to mimic the structural and mechanical properties of natural valves, but electrospinning scaffolds are less efficient to fabricate and have limited ability to create complex 3D structures (140). The Xeltis graft is a pulmonary artery valved conduit made from a range of synthetic materials, including a wall made of polycaprolactone and a valve made of polycarbonate, with modifiable mechanical properties and biodegradability, and has demonstrated good and durable hemodynamic performance in preclinical studies with no stenosis or obstruction or severe regurgitation 2 years after implantation (141). Compared with the histology of Hancock conduit, significant calcification was rarely observed in Xeltis, while Hancock formed more neointimal thickness with the calcification of aortic root (142). 3D bioprinted trilobular valved conduits based on hyaluronic acid methacrylate and gelatin methacrylate have been successfully implanted in mouse models and have shown high cell survival and remodeling potential (143). But 3D printing geometrically complex structures with similar biological behavior to natural tissues remains a great challenge, and ideal scaffold materials and conduit fabrication methods remain an area for continued effort and innovation. Current research advances in tissue engineering scaffolds are mostly limited to preclinical trials (144).

The clinical use of homograft valved conduits is widespread with good long-term outcomes. Although there are still some limitations, with the development of cardiac surgery and other disciplines, research is continuously exploring the best ways to improve the efficacy of homograft valved conduit operation by improving preservation methods, using low-dose immunosuppressive agents, and matching ABO and HLA antigens to mitigate post-transplant immune reactions to extend the life of homograft valved conduits. The development of genetic engineering technology is expected to induce immune tolerance and mitigate immune response through molecular biology techniques. It is also expected that the construction of recipient cellularized tissue-engineered conduit by implanting recipient-derived endothelial cells on decellularized valved conduits or degradable synthetic materials will be an ideal biologic valved conduits substitution for heart disease treatment.

## Author Contributions

YH wrote the text, performed the literature search, and review under guidance from YC. JS reviewed the text. YC and JS revising the manuscript. All authors contributed to the article and approved the submitted version.

## Conflict of Interest

The authors declare that the research was conducted in the absence of any commercial or financial relationships that could be construed as a potential conflict of interest.

## Publisher's Note

All claims expressed in this article are solely those of the authors and do not necessarily represent those of their affiliated organizations, or those of the publisher, the editors and the reviewers. Any product that may be evaluated in this article, or claim that may be made by its manufacturer, is not guaranteed or endorsed by the publisher.
